# Defining the Pathways Underlying the Prolonged PR Interval in Atrioventricular Conduction Disease

**DOI:** 10.1371/journal.pgen.1003154

**Published:** 2012-12-06

**Authors:** Jerry Curran, Peter J. Mohler

**Affiliations:** The Dorothy M. Davis Heart and Lung Research Institute and Departments of Internal Medicine and of Physiology and Cell Biology, The Ohio State University Wexner Medical Center, Columbus, Ohio, United States of America; Stanford University School of Medicine, United States of America

Atrial fibrillation (AF) is the most commonly observed arrhythmia, and it is estimated to affect more than 2 million adults annually in the United States alone [1]. The risk of stroke, heart failure, and sudden death are all increased in patients with AF. The frequency of AF increases with age and most often underlies other overt cardiomyopathies or cardiovascular diseases such as ischemic heart disease, congestive heart failure, or hypertension [2], [3]. However, for nearly 70 years AF has also been known to be an inherited, Mendelian disorder [4]. Indeed, work from the Framingham Heart Study reported a significant increase in the risk of AF in patients who had just one parent with AF [5]. The genetic mechanisms that lead to AF vary widely as do the degrees of penetrance [6], [7].

Atrioventricular (AV) conduction disease is characterized by a prolonged PR interval in surface electrocardiogram (ECG) recordings. Importantly, a prolonged PR interval is a strong predictor of AF; and like AF, variability in the PR interval has a genetic component. However, pinpointing the genetic factors underlying AV conduction has been wrought with difficulty and remarkably little is known about the genes leading to increased susceptibility to PR interval variability. In this issue of *PLOS Genetics*, Lodder and colleagues provide compelling evidence that increased expression of the *Tnni3k* gene (which encodes Troponin I-interacting kinase 3) is significantly correlated with increased PR interval [8].

Previous findings from this research team identified a quantitative trait locus for PR interval variability on chromosome 3 within a mouse model that harbors a variant in the cardiac voltage-gated sodium channel (*Scn5a^1798D/+^*) [9], [10]. In the process of engineering this experimental mouse model, the investigators noted that the ECGs of the parental strains, 129P2-*Scn5a^1798insD/+^* and FVBN/J-*Scn5a^1798insD/+^*, both phenocopied many of the ECG characteristics of human patients harboring the homologous *SCN5A-*1795insD variant, namely conduction defects manifested in the PR interval. These two mouse strains displayed variation in the severity of the PR interval variation, indicating that while the root genetic cause is likely the same, the degree of penetrance diverges owing to differing haplotypes. In their new study, the team cleverly exploits this natural variation to precisely pinpoint the underlying genetic locus, *Tnni3k*, that is shared by the mouse strains and is responsible for PR interval variability. Importantly, the investigators perform in vivo functional studies using mice overexpressing human troponin I-interacting kinase 3 to confirm the role of this gene product in regulating PR interval in atrioventricular conduction.

*Tnni3k* belongs to the MAPKKK family of polypeptides [11]. Interestingly, *PLOS Genetics* recently published work by Wheeler et al. that identified *Tnni3k* as a strong modifier of disease progression in a mouse model of cardiomyopathy [12]. In this work, the evidence implied that *Tnni3k* actively participates only in the stress response pathways within the heart, as mice with minimal *Tnni3k* expression at baseline showed no cardiac abnormalities. Furthermore, and importantly, *Tnni3k* expression is restricted to cardiac tissue. Taken together, this evidence increases the prominence of *Tnni3k* as a potential therapeutic target for multiple cardiac electrical and structural pathologies.

**Figure 1:**
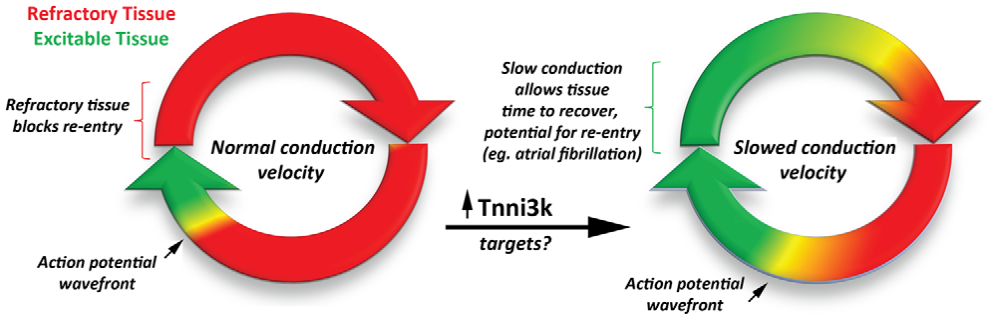
The development of a reentry circuit due to slowed action potential velocity. Increased expression of Tnni3k (and downstream targets) slows conduction velocity of the propagating action potential. Slowed conduction, in turn, favors the formation of stable reentrant propagation, a common mechanism for sustained arrhythmia.

While the genetic and in vivo findings are compelling, the new data from Lodder and colleagues raises questions regarding the exact functional role(s) that *Tnni3k* polypeptides play in the heart (Figure 1). In this study, phosphorylation targets of this kinase were not identified. Indeed, our current understanding of how this kinase functions is broadly lacking. As this work is focused upon AV conduction disease, the authors appropriately speculate that gap junctions and/or cardiac ion channels involved in action potential propagation may be direct targets of Tnni3k. Investigations that identify the phosphorylation targets of Tnni3k are warranted. Furthermore, a clear link to human heart disease has yet to be established: does Tnni3k modify the progression towards heart failure or regulate PR interval duration in conduction diseases as it is now known to in mice? While *Tnni3k* expression is expected to be cardiac-specific, its particular distribution within the heart is unknown. Establishing differential expression in the various chambers, structures (i.e., sinoatrial and atrioventricular nodes), or tissues will further inform us on the functional role(s) of *Tnni3k*.

In summary, Lodder et al. have provided strong evidence that *Tnni3k* regulates PR interval variability in a mouse model of atrioventricular conduction disease. Importantly, this new work highlights the potential for original insights into gene function through creative, varied genetic and physiological approaches, leading to a more educated translation of basic science into new therapeutic targets.
